# Peak cap stress calculations in coronary atherosclerotic plaques with an incomplete necrotic core geometry

**DOI:** 10.1186/s12938-016-0162-5

**Published:** 2016-05-04

**Authors:** Annette M. Kok, Lambert Speelman, Renu Virmani, Antonius F. W. van der Steen, Frank J. H. Gijsen, Jolanda J. Wentzel

**Affiliations:** Department of Cardiology, Biomedical Engineering, Erasmus MC, Rotterdam, The Netherlands; CVPath, Gaithersburg, MD USA; Department of Imaging Physics, Delft University of Technology, Delft, The Netherlands

## Abstract

**Background:**

Stress calculations in atherosclerotic coronary vulnerable plaques can aid in predicting coronary cap rupture. In vivo plaque geometry and composition of coronary arteries can merely be obtained via intravascular imaging. Only optical driven imaging techniques have sufficient resolution to visualize the fibrous cap, but due to limited penetration depth deeper components such as the backside of the necrotic core (NC) are generally not visible. The goal of this study was to investigate whether peak cap stresses can be approximated by reconstructing the backside of the NC.

**Methods:**

Manual segmentations of coronary histological cross-sections served as a geometrical ground truth and were obtained from seven patients resulting in 73 NCs. Next, the backside was removed and reconstructed according to an estimation of the relative necrotic core thickness (rNCt). The rNCt was estimated at three locations along the NC angle and based on either group averaged parameters or plaque specific parameters. Stress calculations were performed in both the ground truth geometry and the reconstructed geometries and compared.

**Results:**

Good geometrical agreement was found between the ground truth NC and the reconstructed NCs, based on group averaged rNCt estimation and plaque specific rNCt estimation, measuring the NC area difference (25.1 % IQR 14.0–41.3 % and 17.9 % IQR 9.81–32.7 %) and similarity index (0.85 IQR 0.77–0.90 and 0.88 IQR 0.79–0.91). The peak cap stresses obtained with both reconstruction methods showed a high correlation with respect to the ground truth, r^2^ = 0.91 and r^2^ = 0.95, respectively. For high stress plaques, the peak cap stress difference with respect to the ground truth significantly improved for the NC reconstruction based plaque specific features (6 %) compared to the reconstruction group averaged based (16 %).

**Conclusions:**

In conclusion, good geometry and stress agreement was observed between the ground truth NC geometry and the reconstructed geometries. Although group averaged rNCt estimation seemed to be sufficient for the NC reconstruction and stress calculations, including plaque specific data further improved stress predictions, especially for higher stresses.

## Background

The main cause of myocardial infarction is coronary plaque rupture. Not all coronary plaques will rupture; therefore it is of imminent importance to distinguish rupture prone plaques from stable plaques. Pathological studies showed that rupture prone plaques consists of a large necrotic core (NC), a thin fibrous cap and often are positively remodeled [21]. However, geometric and compositional information appeared to be insufficient to predict cardiovascular events, therefore new rupture risk parameters should be investigated [23].

From a biomechanical point of view, plaque rupture occurs when the stresses in the cap exceed the strength of the cap. Mechanical studies have shown that stress is an important indicator for plaque vulnerability [10, 24, 26]. Therefore, determining stresses in a cap can aid in predicting cap rupture. A common technique to calculate stresses is by using finite element analysis (FEA). In order to accurately calculate these stresses, blood pressure, material properties, and geometric factors are required. The most important geometric factor influencing peak cap stress (PCS) predictor is cap thickness, but also lumen size and shape and NC size and shape [1, 2, 7, 8, 16, 19].

To capture plaque geometry and composition in vivo, intravascular imaging is required. The only imaging techniques with sufficient resolution to accurately visualize the cap thickness are optical driven, such as optical coherence tomography. However, necrotic core tissue highly attenuates light, and thereby limits the penetration depth. Consequently, the NC geometry can become obscured, and thus is often only the front side of the NC visible. In order to allow FEA the complete geometry of the NC is essential.

The goal of this study was to investigate whether peak cap stresses can be approximated by reconstructing the backside of the NC. Therefore, we used a three-step approach: (1) a model was generated based on the histology data to estimate the NC thickness at several locations along the NC angle, (2) the backside of the NC was artificially removed and reconstructed based on characteristics of the NCs, (3) stresses and geometries of the plaque containing the reconstructed NC geometries were compared with the plaque containing the ground truth NC.

## Methods

In order to reconstruct the backside of the NC, MATLAB (version 2014a, Mathworks Inc., Natick, MA, USA) was used. Six steps were taken: (1) manual segmentation of the coronary plaque geometry from histology (‘ground truth’), (2) geometrical characterization was performed of all the plaques and NCs: to determine the group average (relative) NC thickness at the center (50 %) of the NC angle (midcap) and ±25 % of the NC angle (sidecaps) and corresponding plaque specific properties: cap thickness, NC angle, and intima-media thickness (IMT) were determined, (3) plaque specific relation between the relative NC thickness (rNCt) and plaque specific properties was determined with a generalized estimation equation (GEE) model, (4) the backside of the NC geometry was removed and thereafter reconstructed based on group averaged rNCt estimation and a plaque specific rNCt estimation, (5) geometry assessment: the reconstructed NC geometries were compared with the ground truth geometry, and (6) the effect of the reconstruction on the PCS calculations was evaluated. All steps are explained in more detail below.

### Histology

Histology was used to obtain the NC geometry in atherosclerotic plaques to serve as the geometrical ground truth. It is important to obtain representative plaque cross-sections. Therefore, we used coronary plaque data of seven patients who died from severe coronary artery disease. The specimens were obtained from CVpath (CVPath Institute, Inc., Gaithersburg, MD, USA). Only those cross-sections that contained at least one NC were included. The coronary arteries were perfusion fixed in formalin at 100 mmHg prior to the histological preparation. Each consecutive cross-section had a distance of at least 200 μm to the next cross-section in the longitudinal direction. In total, 54 cross-sections (5 μm in thickness) were obtained from 13 different arteries of which 31 plaques included one necrotic core and 21 contained two NCs. When multiple NCs were present in a cross-section, the NCs were treated separately; this resulted in 73 plaque geometries. A Modified Movat pentachrome staining was used to identify different plaque components. The lumen, NC, intima, media and adventitia layer were manually delineated. The ground truth contours were finalized during a consensus meeting.

### Plaque and necrotic core characterization

**Fig. 1:**
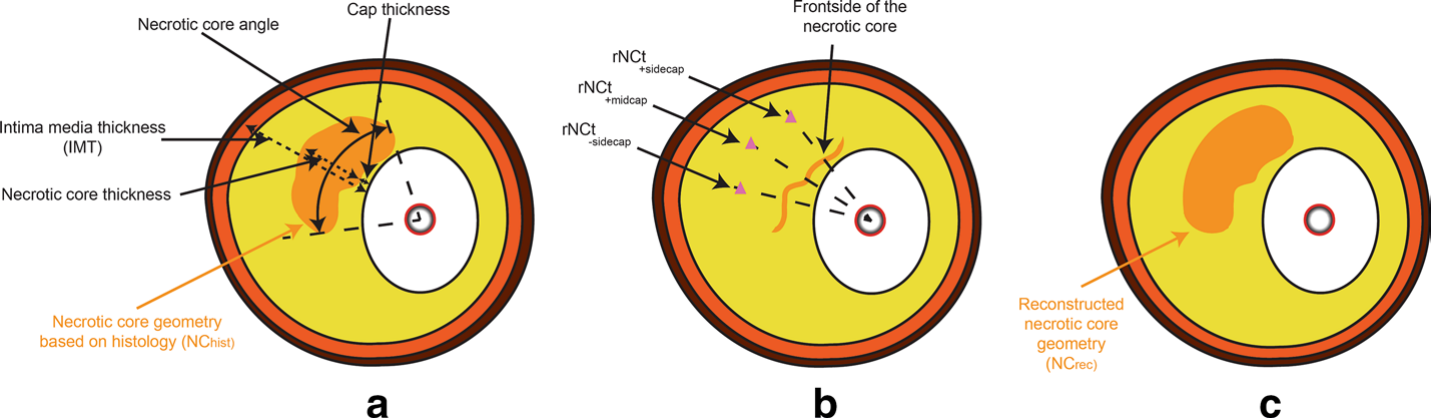
A schematic overview of the coronary plaque (**a**) and the corresponding reconstruction methodology (**b** and **c**). The adventitia (*brown*), media (*dark orange*), intima (*yellow*) and the necrotic core (*orange*) were obtained by segmentations of the ground truth. **a** overview of the plaque specifics properties, **b** the backside is removed and the relative NC thicknesses (rNCt) (*pink triangles*) were calculated for the mid and sidecaps, and **c** the reconstruction of the necrotic core

Geometrical characterization of the atherosclerotic plaque was performed by determining the geometrical properties of the plaque and the NC, see Fig. 1a. First, the minimum cap thickness and the front side of the NC were determined from the perspective of the lumen center, see Fig. 1b. Second, the NC angle was determined as the angle of the front side of the NC geometry, also with respect to the center of the lumen. Third, at three locations along the NC angle, the NC thickness was determined: at the center (50 %) of the NC angle (midcap) and ±25 % of the NC angle (±sidecap). Also, the cap thickness (capT) and IMT were determined at these corresponding positions, see Fig. 1a.

### Group averaged necrotic core thickness estimation

From the plaque and NC characterization we obtained group averaged NC thicknesses at three different locations: midcap and both sidecap positions. To make it robust for all plaque sizes, these NC thicknesses were divided by their corresponding IMT hereafter referred to as relative NC thickness (rNCt). The median of the whole plaque group was calculated for the midcap position and the median was calculated for both sidecap positions. These two rNCt were then used in the NC reconstruction, this method is hereafter referred to as the group averaged method.

### Plaque specific necrotic core thickness estimation

Since, we assume that the NC thickness depends on plaque characteristics, we developed a method that estimates the NC thickness on a plaque specific level. Generalized estimation equation (GEE) model was used to determine a relationship between NC thickness and plaque specific parameters. In order to determine if each parameter in the model significantly contributed, the covariance matrix of β was determined. This was done by a method previously described by Liang and Zeger et al. [15]. To also make the GEE model more robust, the rNCt was calculated. The plaque specific geometrical parameters used as input for the GEE model consist of NC angle, IMT, and cap thickness. We assume that the rNCt is symmetrical around the center of the NC angle, therefore two relationships were derived; one for the midcap and one for the combined sidecap positions. This relationship was defined as:

1 $$\begin{aligned} rNCt_{i} &= \beta_{0,\ i} + \beta_{NC\,\, angle,\ i} \cdot \left( {NC\, angle\left[ \rm{{rad}} \right] } \right) + \beta_{IMT,\ i} \cdot \left( {IMT\left[ {\upmu \rm{m}} \right] } \right) \nonumber\\ & \quad + \beta_{capT,\ i} \cdot \left( {capT\left[ {\upmu \rm{m}} \right] } \right) \end{aligned}$$

with i indicating the relation at midcap or ±sidecap and *β* the constants determined by the GEE model. The GEE model was corrected for correlation amongst cross-sections within an artery [20]. Next, for each plaque the rNCt at the midcap and side cap positions was determined with the GEE model.

### Reconstruction

**Fig. 2:**
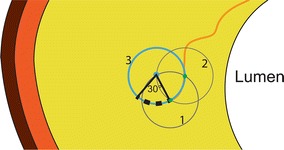
Schematic illustration of the creation of the side of the NC using part of a *circle*. To calculate the center point of the *circle* used for creation of the side of the NC a combination of *circles* was used. The center of *circle 1* was located at the edge of the necrotic core. One point in *circle 1* intersects the cap, and this point is used as center point for *circle 2*. Then the two *circles* intersect at two locations, and the intersection most distant from the lumen was the center point of the final *blue circle* (*3*) for the side of the necrotic core (*blue dot*). Together with the empirically determined radius and angle the side can be reconstructed, see the *black dotted line*

Optical driven imaging techniques suffer from fatty tissues, since they highly scatter and absorb light, therefore limiting visualization beyond the NC. Thus, only the front side of the NC can be visualized reliably. To account for this only the front line of the NC was kept. This was done by drawing a line from the lumen center to each point in the NC. If the point was not the first intersection it was removed, in this way the backside of the NC was removed. The NC reconstruction was performed based on the group average rNCt estimations and the plaque specific rNCt estimation. The rNCt (at midcap and sidecaps) were converted to the absolute NC thickness and were placed at the corresponding positions. From histology we observed that NCs often have rounded features at the edges. Therefore, a part of a circle was attached to the edges of the NC, this also prevents geometric discontinuities in the reconstructed NC. The radius and angle of this part of the circle was empirically determined by optimizing the overlapping areas of all the reconstructed NCs with the ground truth areas. The optimized value for the angle was 30 [°] and the radius was dependent on the NC angle: 0.14 × NC angle [rad], these values were used for all the NC reconstructions. A visualization and description of how the center point of this circle was obtained is given in Fig. 2. Hereafter the backside is closed with the attached part of the circle by forcing the polynomial function to go through the side of the attached part of the circles (Fig. 1c). In case the polynomial function intersects the media layer (n = 20 for the group average rNCt, n = 8 for the plaque specific rNCt estimation), the NC area outside the intima is removed. Since it is not physiological that the NC is attached to the medial layer, a small layer was removed from the NC. In order not to alter the predicted geometry this layer was kept very small (10 μm).

### Geometry analysis

To investigate to what extent the reconstructed NC geometry corresponds with the histology based NC geometry, two measures were used: (1) the similarity index (SI) and (2) the relative difference in NC area, ΔA(%). The SI is a measure for overlap, defined as:

2 $$SI = \frac{{2 \cdot \left\{ {A_{GT} \mathop \cap \nolimits A_{rec} } \right\}}}{{A_{GT} + A_{rec} }}$$

with A_GT_ the area of the ground truth NC geometry and A_rec_ the area of the reconstructed NC geometry. To quantify mismatches in area the ΔA(%):

3 $$\Delta A\left( \% \right) = \frac{{\left| {A_{rec} - A_{GT} } \right|}}{{A_{GT} }} \cdot 100\;\%$$

was used, with A_GT_ the area of ground truth NC and A_rec_ the area of the reconstructed NC.

### Wall stress calculation

**Fig. 3:**
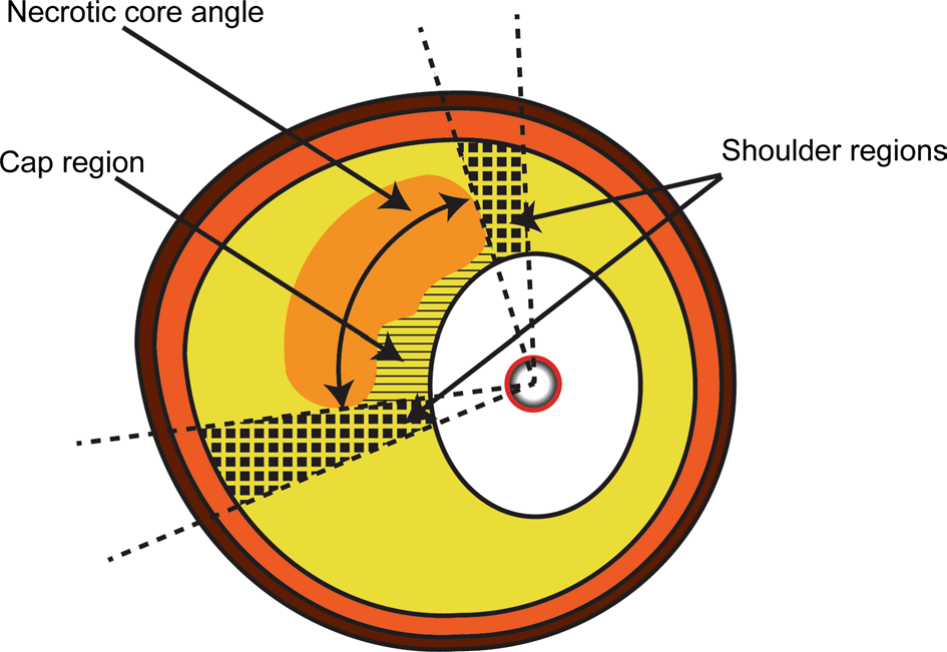
A schematic illustration of the regions were the peak cap stress (PCS) is determined. The PCS was defined as the maximum von Mises stress located in either the cap region (*striped area*) or in the shoulder regions, 15° adjacent to the NC (*blocked area*)

Contours of the plaque with the ground truth NC and the reconstructed NC were imported and meshed in Abaqus for FEA (version 6.13, Dassault Systemes Simulia Corp., Providence, RI, USA). All material properties were assumed to be homogeneous and incompressible. Neo-Hookean material models for all components were used, as was done in similar parametric studies [3, 12, 22]. The mechanical properties of the components are listed in Table 1. To restrain rigid body motion, a compressible soft buffer around the model was used and the outer contour of the buffer was fully constrained. The material properties of the buffer are also listed in Table 1. Plaque stresses were computed by solving the mass and momentum equations. Linear tetrahedral elements were used with at least seven elements at the minimum thickness of the cap. One simulation of a 2D plaque took approximately 4 h with typically 1 × 10^6^ elements on a standard desktop computer. Due to pressure fixation in the histology process, the histology based geometry was not stress free. Therefore, initial stresses up to 100 mmHg were calculated with the backward incremental method [22]. Subsequently, a systolic blood pressure of 140 mmHg was applied. PCS was defined as the maximum von Mises stress in the cap (defined as intima in front of the NC) and at the shoulders of the cap; defined as the area 15° adjacent to the cap, see Fig. 3 [1]. Peak cap stresses were calculated and analyzed for the intima of the geometry containing the ground truth NC from histology (PCS_GT_), the reconstructed NC based on group average rNCt estimation (PCS_GA_) and the NC based on plaque specific rNCt estimation (PCS_PS_). The distance between the PCS location of the ground truth and the reconstructed plaque was determined. Hereafter co-localization was determined based on visual inspection. If the PCS was delocalized, displacement was classified as (1) shift from one side to the other side of the NC (2) slight delocalization or (3) translocation from the lumen to the NC side or vice versa.

**Table 1:** Material properties of the buffer and different components in the plaque

Material	E-modulus (kPa)	Poisson ratio (−)	C_10_ (kPa)	D_1_ (−)	Reference
Intima	1000	0.498	166.7	1e−5	[11]
Media + adventitia	1500	0.498	250	1e−5	[11]
Necrotic core	6	0.498	1	1e−5	[17]
Buffer	60	0.45	10	0.02	[6]

C_10_ and D_1_ are constants to describe the Neo-Hookean model as used by Abaqus, representing the shear and bulk modulus as: G = 2×C_10_ and K = 1/D_1_

### Statistics

The data was tested for normality with a Kolmogorov–Smirnov test. If the data was normally distributed, mean and standard deviation are given and differences between data were tested with a paired t-test. Otherwise, the median and interquartile range were given and a paired non-parametric test (Wilcoxon signed ranktest) was used to test for significant differences. Statistical significance was considered as p < 0.05. The SI and ΔA% were analyzed for the geometry of the ground truth NC (NC_GT_) vs. the reconstructed NC based on group averaged rNCt (NC_GA_), and the reconstructed NC based on the plaque specific rNCt (NC_PS_). Good similarity index was assumed to be at least SI > 0.8. Linear regression analysis was performed to compare peak wall stress of the different models.

## Results

On average, the 73 plaques had a minimum cap thickness of 0.20 mm (0.09–0.40 mm) and NC angle of and 54° (35–75°). At the midcap location, the cap thickness, IMT, and absolute NC thickness were 0.30 mm (0.13–0.53 mm), 1.05 mm (0.89–1.29 mm), and 0.46 mm (0.27–0.54 mm), respectively. For the sidecaps, the cap thickness, IMT, and absolute NC thickness were 0.31 mm (0.16–0.54 mm), 1.03 mm (0.84–1.24 mm), and 0.36 mm (0.21–0.51 mm). The median of the relative NC thicknesses at midcap and sidecap positions were 0.40 and 0.35, respectively.

The GEE model showed a significant correlation between the estimated rNCt and the ground truth rNCt: at midcap (r^2^ = 0.47) and at sidecap (r^2^ = 0.44), see Table 2. All parameters (cap thickness, IMT and NC angle) were found to have a significant contribution for predicting the rNCt at both positions.

**Table 2:** Generalized Estimation Equation (GEE) parameters with their standard errors

	*β*_0_	$\beta_{necrotic \,\,core \,\,angle}$	*β*_*IMT*_	$\beta_{capT }$	r^2^	p
rNCt_midcap_	187 ± 55.4	63.3 ± 26.6	0.29 ± 0.07	−0.51 ± 0.08	0.47	<0.05
rNCt_±sidecap_	175 ± 46.4	130 ± 19.8	0.18 ± 0.05	−0.42 ± 0.06	0.44	<0.05

Predictive for the relative necrotic core thickness (rNCt), the r^2^ and p value of the regression line between the actual rNCt value and the GEE model estimated value rNCt

All parameters with corresponding standard deviations are ×1000-fold

**Fig. 4:**
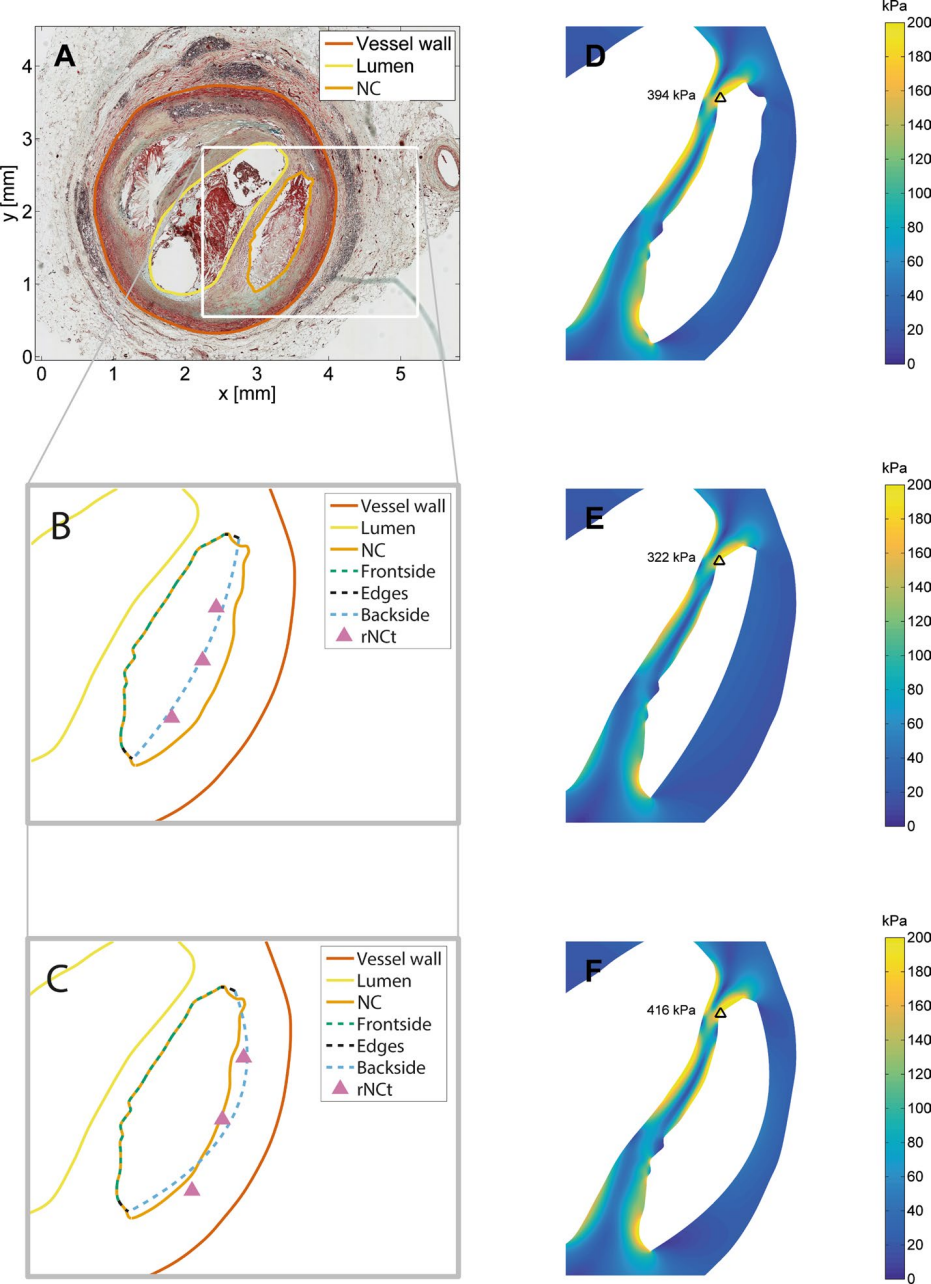
From the histology image to stress calculations. **A** Cross-sectional slice from histology with the vessel wall, lumen, and necrotic core already delineated. **B**–**F** are zoomed versions of (**A**) with necrotic core reconstruction based on the group averaged NC data (**B**) and with necrotic core reconstruction based on the plaque specific (**C**) rNCt estimation methods. **D**–**F** are the wall stresses of the plaque containing the necrotic core geometry from the ground truth (**D**), reconstruction of the necrotic core based on group averaged data (**E**) and reconstruction of the necrotic core based on plaque specific (**F**) method and the *black triangles* indicate the location of the peak cap stress

Figure 4A–C shows a typical example of the segmentation and reconstruction of the NC. The NC reconstruction based on group average (Fig. 4B) underestimated (SI = 0.83) the NC area, whereas the plaque specific (Fig. 4C) NC reconstruction predicted (SI = 0.93) the NC area better. For all plaques, the NC_GA_ matched the NC_GT_ well with a SI and ΔA % of 0.85 (0.77–0.90) and 25.1 % (14.0–41.3 %), respectively. The NC_PS_ matched the NC_GT_ even better with a SI and ΔA % of 0.88 (0.79–0.91) and 17.9 % (9.81–32.7 %), respectively. The NC geometries based on plaque specific rNCt had significantly better SI and ΔA% (p < 0.01) opposed to the NC geometry that were reconstructed solely based on group averaged rNCt.

**Fig. 5:**
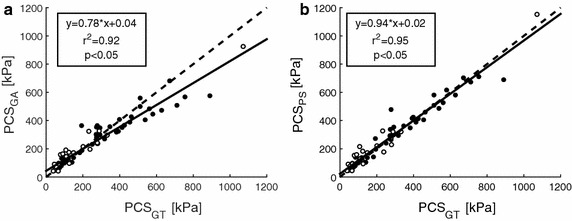
The peak cap stress (PCS) of 73 plaque models from ground truth (histology) (*x-axis*) versus the peak cap stress of the reconstructed necrotic core geometry (*y-axis*) are depicted. In **a** the PCS based on the NC reconstruction using group averaged rNCt estimation, PCS_GA_, and **b** PCS based on the NC reconstruction using the plaque specific rNCt estimation, PCS_PS_. The *closed circles* indicate the cases for which the locations of the peak cap stresses was not shifted with respect to the ground truth, whereas the *open circles* did shift

For the typical example also the corresponding stress distribution in the fibrous cap of the ground truth geometry, and the reconstructed geometries are depicted in Fig. 4D–F. The PCS_GA_ and PCS_PS_ for this example showed 18 and 6 % PCS difference with respect to the ground truth PCS. The absolute PCS values were 192 kPa (82.1–355 kPa) for the ground truth, 192 kPa (96.0–354 kPa) for the group average method and 196 kPa (95.6–369 kPa) for the plaque specific method. The overall difference in PCS between PCS_GT_ vs. PCS_GA_ was 15 % (5–25 %) and PCS_GT_ versus PCS_PS_ was 8 % (5–23 %). For all plaques, no significant difference was found in peak cap stresses between both reconstruction methods and the ground truth, only PCS_GA_ versus PCS_PS_ showed a significant difference (p < 0.05). The correlation between the PCS of the ground truth and the PCS of the reconstructed geometries are shown in Fig. 5a, b. Both the PCS_GA_ and PCS_PS_ showed a high correlation with PCS_GT_, r^2^ = 0.91 and r^2^ = 0.95, respectively. The PCS_GT_ vs. PCS_GA_ showed a higher offset and lower slope compared to the regression line obtained with the PCS_GT_ vs. PCS_PS_.

In Fig. 5a, there seems to be a visual cut-off value at 300 kPa were the group averaged method seems to underestimate the peak cap stresses. Coincidentally, this 300 kPa threshold is also mentioned in literature as a rupture risk threshold [5]. In order to investigate if the *high* PCS_GT_ were better estimated for the PCS_PS_ than the PCS_GA_, the cross-sections were divided based on the PCS in a low (<300 kPa) and high stress (>300 kPa) group. Next, for both groups the PCS differences with respect to the ground truth were calculated. For the plaques with PCS_GT_ below 300 kPa both reconstruction methods showed 15 % difference in PCS (p > 0.05) compared to the ground truth. Interestingly, for the plaques with relative high PCS_GT_ (>300 kPa), the PCS difference was 16 % for the group averaged rNCt and only 6 % for the plaque specific rNCt based reconstruction (p < 0.05).

The distance between the locations of the PCS in the ground truth compared to the reconstructed geometries were evaluated. Since the distance between the location of the PCS_GT_ and PCS_GA_ was not different from the distance between the location of PCS_GT_ and PCS_PS_ (p = 0.29), only the distances between PCS_GT_ and PCS_PS_ are hereafter listed. In 67 % (n = 49), the location of the PCS_PS_ was similar as the location of the PCS_GT_, with a distance of 4.13 μm (2.66–17.6 µm), indicated by the closed circles in Fig. 5. For the remaining 33 % of the data (n = 24), the location of the PCS was not similar and is depicted in Fig. 5 by the open circles. The shift in PCS was (1) from one side of the NC to the other side (n = 9) with a distance of 0.99 mm (0.72–1.47 mm) or (2) at the same side (n = 4) with a distance of 0.21 mm (0.07–0.66 mm) or (3) translocated from the lumen side towards the front side of the NC or vice versa (n = 11) with a distance of 0.92 mm (0.24–1.32 mm). For plaques that showed a shift in the peak stress location, the difference in PCS compared to the ground truth was larger (35 %) than for plaques with the same peak stress location (11 %). The PCS_PS_ that were not at the same locations as the PCS_GT_ had stresses mostly below 300 kPa and represent the more stable plaque: 160 kPa (93.9–226 kPa), respectively. For the PCS_PS_ at the same location as the ground truth the stresses had a higher variation: 281 kPa (102–392 kPa).

## Discussion

In this study, we analyzed the effect of the reconstruction of the missing NC backside with respect to geometry and stress calculations. The backside of the NC can be obscured due to limited penetration depth of in vivo light based imaging in coronary arteries. Histological cross-sections (‘ground truth’) were used to characterize the plaque and to reconstruct the NC geometry. Reconstruction was performed by determining the relative NC thickness (rNCt) at three locations along the NC angle. The rNCt was either based on a group average or plaque specific parameters. Subsequently, the reconstructed NC geometries and the corresponding plaques from both methods were compared with the ground truth regarding the geometry and PCS. The following are the two *main findings* of this study: (1) high agreement was found between the NC geometries and the corresponding peak cap stresses of the reconstructed NCs and the ground truth, (2) reconstructing the NC based on average NC data resulted in underestimated peak cap stresses for high stress plaques only, whereas including plaque specific data for the NC reconstruction improved the stress prediction for these high stress plaques.

In terms of PCS prediction in the low PCS group (<300 kPa), representing the more stable plaques, both NC reconstruction methods performed equally well, resulting both in 15 % difference in PCS. This is relative low compared to changes in PCS in idealized models due to cap material properties (55–200 %) [2]. In patient specific plaque geometries, the PCS showed to have two independent predictors: cap thickness and lumen curvature [1]. In the current study, the cap thickness and lumen geometry were unchanged, which may explain the relative good prediction of the peak cap stresses with the NC reconstruction methods.

A significant improvement in PCS was found for high PCS group (>300 kPa) using the plaque specific NC reconstruction method. This could be explained by the fact that an increase in NC area affects the PCS more in thin compared to thick fibrous caps [2]. Thus, it is likely that for high stress plaques, which have in general thin caps, a more precise reconstruction of the NC results would result in more accurate PCS prediction. Indeed, our data showed that for the plaques with a thin cap (<0.20 mm), (a) the corresponding SI and ΔA% significantly improved using plaque specific NC reconstruction method (SI: 0.83 vs 0.87 and ΔA% 25 vs. 14 %) (b) the PCS estimation improved with respect to PCS_GT_ when using plaque specific information (difference PCS_GA_ 14 % and PCS_PS_ 7 %) whereas plaques with a thick cap did not show a significant improvement (15 vs. 18 %).

For in vivo PCS computations in (vulnerable) coronary plaques, visualization of the cap is needed. The only possibility to obtain the cap thickness is with optical driven imaging techniques (e.g., optical coherence tomography, OCT), but it has limited penetration depth. Therefore the deeper lying tissues will become obscured, causing incomplete geometries for stress calculations. In this study, on purpose we investigated the influence of the reconstruction of the NC only. Using histology as ground truth enabled us to isolate the effects of reconstruction of the NC from possible other factors that can influence the PCS calculation. Logically, if the NC is obscured also the deeper laying vessel wall is missing. In a recent study it was shown that the vessel area in coronary arteries can be approximated by interpolating the non-obscured vessel wall [13]. Thus when moving forward to a clinical application the latter technique should be combined with the reconstruction of the NC to enable in vivo PCS calculations in coronary arteries.

Another attractive feature of OCT is that macrophages can be imaged. Extensive research showed that macrophages are linked with plaque instability [14, 18, 25], therefore macrophages can be interpreted as a surrogate marker of cap strength. Co-localization of PCS and presence of macrophages might be instrumental in risk prediction. In that respect not only the PCS is of importance, but also the location. In this study a number of the PCS locations were not co-localized with the PCS_GT_ and thus might have consequences for risk prediction. However, most of the plaques with incorrect PCS locations showed low peak cap stresses, representing the more stable plaque.

Histological processing and variations in the manual segmentation might affect the ultimate geometry of the plaque and NC. We showed in an earlier study that histological processing hardly affects the geometry compared to the in vivo situation (Groen et al. 2010). However, since the model is tuned to this data-set, it is conceivable that variations because of the manual delineation and histological processing has some influence on the outcome of this model. We expect that the peak cap stress is minimally influenced, since we showed that the peak cap stress are hardly influenced by the exact geometry of the backside of the NC, and we are therefore confident that the minor impact of intra- and inter-observer variability on the model has also minor impact on the final stress. Therefore, it will not change the main conclusion of this study. Despite the relative large sample size of NCs (n = 73), the geometrical variation in the histology contours might not completely represent the whole population of NCs, due to the limited number of patients (n = 7). Little data is available on the geometrical variation of NC dimensions (e.g., lipid angle and NC thickness at midcap and sidecaps of the lipid angle). In a study by Gardner et al. [9], lipid cores were only assessed in plaques with a lipid core >60° in circumferential extent, >200 μm thick lipid core, and a mean fibrous cap thickness <450 μm. They found a median minimum cap thickness of 164 μm (IQR 101–243 μm), a median lipid angle 102° (IQR 77–132°), and a median NC thickness of 448 μm (IQR 315–565 μm) [9]. The NC thickness in our study showed a similar range (461 μm, IQR 268–0.543 μm), however, our median lipid angle was lower and showed less variation: 54° (IQR 35–75°). This might be not so accurate to the estimation of the NC thickness and wall stress calculations for larger angles. However, no significant differences were found in PCS between plaques with a NC angle below or above the median NC angle of 60.9° (20.6 and 22.6 %).

In the stress calculations of this study relative simple material models were chosen for the plaque components. In diseased tissue there are usually extra collagen fibers present to absorb higher stresses. Ideally, tissue nonlinearity and anisotropy should be taken into account. However, to the knowledge of the authors, experimental data representing anisotropy in coronary arteries is not available. Although in carotid arteries this data is available, the dispersion range is so high that modeling with isotropic model could suffice [4]. In addition to anisotropy and the geometric factors other factors will also influence the absolute values of PCS, such as, luminal pressure, initial stress, residual stress, 2D vs 3D assumptions, and material properties. The latter, might be the most important one, since it was shown that in an idealized geometry the PCS could change 200 %, just by changing the material properties of the intima. Since in this study in all models, the ground truth and reconstructed, the same material properties were used, no differences in absolute PCS based on the material properties may be expected. Therefore, conclusions on the reconstruction methodology and resulting differences in PCS still hold. However, when performing plaque specific risk analysis, nonlinearity and anisotropy will definitely impact the stresses.

In this study, we only retain the front line of the NC such that it resembles the optical driven imaging techniques. Another approach would be removing the NC behind a certain distance from the lumen. In this way, other features such as the edges of the NC and NC area change. It would be interesting to investigate to what extent these edges and area change affect the PCS calculation. However, it is arguable whether these methods will affect our results, since we already observed that a significant area difference did not result in a significant PCS change between the reconstruction methods. Another approach to refine our method in future work is to improve the GEE model by categorizing NC cores based on, size, shape, and location in the vessel wall. This refinement will probably improve the predictive value of the GEE model. However, we already showed that the PCS estimation between the group averaged and plaque specific method does not differ that much. Therefore, it is arguable whether this categorizing method will show an improvement in cap stress.

## Conclusions

In conclusion, this study showed that reconstruction of the backside of a NC can be performed using either group averaged NC data or plaque specific NC data. A high correlation was found between peak cap stresses as obtained with the ground truth geometry and the peak cap stresses of both reconstruction methods. Although using group averaged NC data for the reconstruction of the backside of the NC performs well for the stress calculations, including plaque specific data in the NC reconstruction method improves the stress prediction, especially for high stress plaques.
